# Prebiotic Assembly of Cloverleaf tRNA, Its Aminoacylation and the Origin of Coding, Inferred from Acceptor Stem Coding-Triplets

**DOI:** 10.3390/ijms232415756

**Published:** 2022-12-12

**Authors:** Ilana Agmon

**Affiliations:** 1 Schulich Faculty of Chemistry, Technion-Israel Institute of Technology, Haifa 3200003, Israel; chilana@technion.ac.il; 2 Fritz Haber Research Center for Molecular Dynamics, Hebrew University of Jerusalem, Jerusalem 9190401, Israel

**Keywords:** genetic code, origin of life, prebiotic aminoacylation, stereochemical hypothesis, translation, tRNA evolution

## Abstract

tRNA is a key component in life’s most fundamental process, the translation of the instructions contained in mRNA into proteins. Its role had to be executed as soon as the earliest translation emerged, but the questions of the prebiotic tRNA materialization, aminoacylation, and the origin of the coding triplets it carries are still open. Here, these questions are addressed by utilizing a distinct pattern of coding triplets highly conserved in the acceptor stems from the modern bacterial tRNAs of five early-emerging amino acids. Self-assembly of several copies of a short RNA oligonucleotide that carries a related pattern of coding triplets, via a simple and statistically feasible process, is suggested to result in a proto-tRNA model highly compatible with the cloverleaf secondary structure of the modern tRNA. Furthermore, these stem coding triplets evoke the possibility that they were involved in self-aminoacylation of proto-tRNAs prior to the emergence of the earliest synthetases, a process proposed to underlie the formation of the genetic code. Being capable of autonomous materialization and of self-aminoacylation, this verifiable model of the proto-tRNA advent adds principal components to an initial set of molecules and processes that may have led, exclusively through natural means, to the emergence of life.

## 1. Introduction

**Figure 1:**
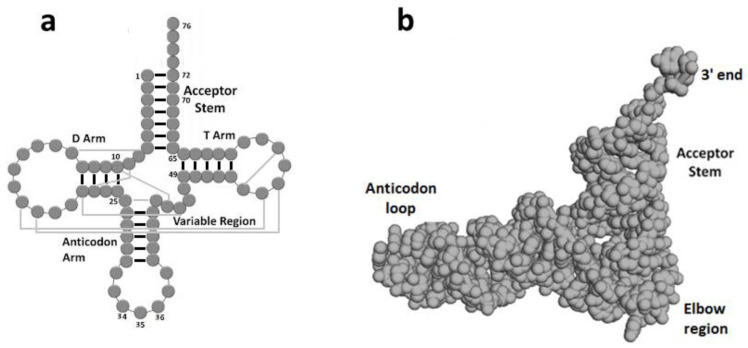
Canonical tRNA (**a**) secondary structure. The TΨC, DHU arms are referred to as T, D arms, respectively. Long range base pairs are sketched in light gray. (**b**) Tertiary structure (tRNA^Ser^ pdb 4V9I).

**Figure 2:**
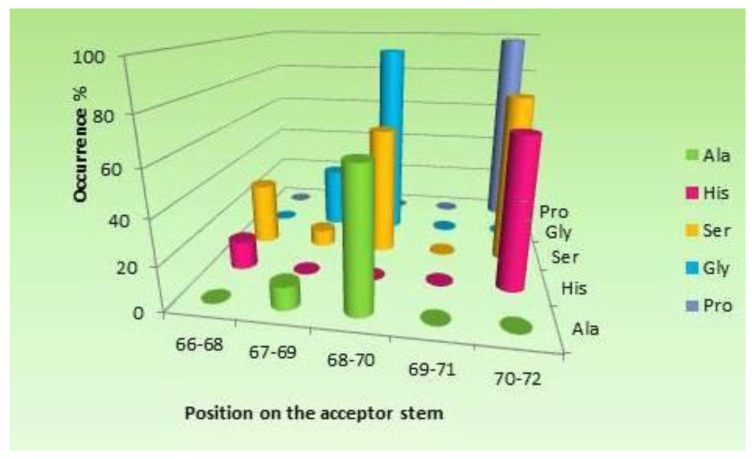
Conservation of coding triplets in the 3′ side of the acceptor stem from five bacterial tRNAs [9]. The identity of the conserved triplets and their percentage of occurrence are given in Figure S1.

tRNA molecules are principal components of the modern translation system, where genetic codes, embedded in mRNA strands, are turned into proteins. Canonical tRNAs have a four-armed cloverleaf secondary structure and an L-shaped tertiary structure (Figure 1a,b). The anticodon (AC) loop carries three nucleotides (positions 34–36) that fully characterize the amino acid esterified to the 3′ end tail. The tRNA molecule therefore possesses the unique capacity of mediating between the mRNA, accommodated on the small ribosomal subunit, and the Peptidyl Transferase Center (PTC), the active site of the large ribosomal subunit, where its cognate amino acid joins the nascent polypeptide. Twenty tRNAs are specifically associated with the 20 canonical amino acids. Their aminoacylation is accomplished by their cognate synthetases, which acquire the identity of the tRNA mainly from the anticodon triplet [1], but also from additional recognition elements classified as ‘‘determinants’’ and ‘‘anti-determinants’’ [2], such as the variable loop of tRNA^Ser^, the discriminator base (N73), and a few nucleotides at defined positions in the acceptor stem [3,4,5]. Nucleotides contained within the first five base pairs from the acceptor stem of certain amino acids were demonstrated to carry sufficient coding information to confer specific aminoacylation, and minihelices derived from these acceptor stems were specifically charged by their cognate synthetases [4,5]. These stem identity determinants, also termed “operational code” [4], were suggested to be remnants of a primordial mechanism for specific aminoacylation of proto-tRNAs [4,6], whereby the initial synthetase, assumed to have had a limited size, could have reached coding information in the stem, adjacent to the 3′ end [4,7,8]. Recently, large scale statistical analysis of sequences from the acceptor-TΨC stem [9], revealed that almost all the stem identity determinants found in the bacterial tRNAs of Ala, Gly, His, Pro, and Ser are contained within their cognate coding triplets, which are extremely conserved in positions 68–70 and 70–72 (Figure 2). These coding triplets, same as the “operational code”, were proposed to be a relic from a prebiotic aminoacylation mode, but one that was controlled by the full stem coding triplets, rather than merely by the discrete identity determinants. Their high evolutionary preservation was attributed to their role as recognition elements in the aminoacylation mode utilized by the modern corresponding synthetases [9] which belong to class IIa [10].

Here, an array of coding triplets, following their pattern in the bacterial acceptor stems of Ala, Gly, His, Pro, and Ser (Figure 2) is used as a building block for composing a proto-tRNA model. Self-assembly of several copies of a single 12-mer RNA oligonucleotide that carry this array, generates in a simple and statistically feasible process, a proto-tRNA with cloverleaf secondary structure, closely compatible with the modern tRNA. The coding triplets conserved in the acceptor stems from the tRNAs of these five amino acids are proposed to have controlled a self-aminoacylation process of the corresponding proto-tRNAs, prior to the emergence of the earliest synthetases. This role is suggested to underlie the formation of the genetic code, while the conserved triplets are assumed to be the fundamental coding triplets, which have only later been used as recognition elements in the anticodon loop. Experimental procedures for verifying the self-assembly of proto-tRNAs and their self-aminoacylation capability are proposed.

## 2. Results

### 2.1. Shape of the Proto-tRNA

**Figure 3:**
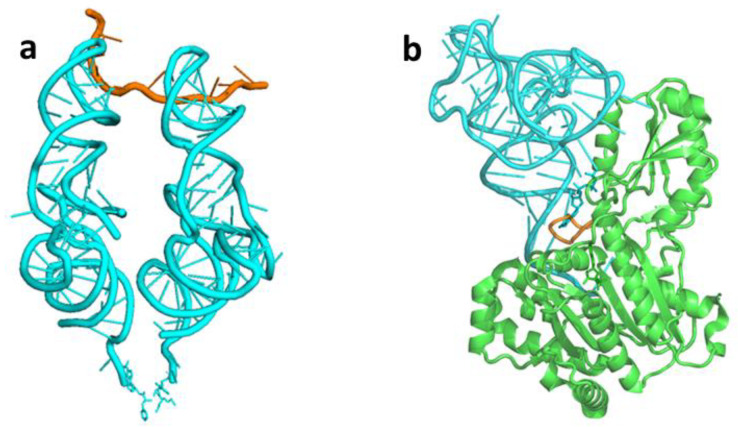
tRNA interactions in current biology: (**a**) 70S ribosome: A-, P-site tRNAs (cyan), attached to adjacent codons on mRNA (orange) (PDB code 1VY4), form a rhombus-like arrangement that prevents bumping of the stems. (**b**) Synthetase interaction with the acceptor stem in the tRNA^His^:HisRS complex (PDB code 4RDX). Motif 2 loop (orange) of HisRS (green) bulges from the catalytic core into the major groove of tRNA^His^ (cyan) acceptor stem.

The evolutionary history of tRNA has gained considerable attention, due to its central role in translation and its linkage to the origin of the genetic code. Hairpins carrying an AC triplet in their loop and having a cognate amino acid attached to their 3′ end were suggested to have been the progenitors of the modern tRNAs. By lining up, side by side, along the RNA string serving as mRNA, such hairpins were proposed to have enabled the formation of the first coded peptides [11,12,13,14,15]. However, the subsequent determination of high-resolution structures of 70S ribosomes complexed with tRNAs base paired to neighboring codons on an mRNA string, demonstrated that such hairpins are bound to collide. The maximal length measured for a codon triplet is 18.2 Å, while the width of the AC stem ranges between 20.6–21.7 Å [16]. The stem’s collision is prevented, in the contemporary ribosome, by a kink in mRNA, generated via its interaction with helix 44 of the small subunit [17]. The kink separates the AC stems of the tRNAs accommodated at the A-, P- sites, and only the perpendicular acceptor-TΨC arms, which are inclined towards each other, can place the two reacting amino acids in the proximity required for peptide bond formation (Figure 3a). Consequently, in case one holds to the premise that the prebiotic codons were nucleotide-triplets, hairpins would have failed to participate in translation. This steric consideration, combined with the conformity to the continuity principle [18], suggests that the first proto-tRNA molecules engaged in primordial translation were already L-shaped.

### 2.2. Initial Coding and Specific Aminoacylation of Proto-tRNAs

Elucidating an evolutionary pathway to the specific aminoacylation of proto-tRNAs is an indispensable step for linking the genetic code with protein synthesis. A feasible mechanism whereby proto-tRNAs could have been self-aminoacylated, would make the initial existence of a charging enzyme redundant, thus eliminate the chicken and egg conundrum where charging the prebiotic tRNA required a synthetase that demanded aminoacylated tRNAs for its synthesis.

Carl Woese’s stereochemical hypothesis [19], which assumes affinity of certain amino acids towards their cognate codon or AC triplets, can form the basis of a prebiotic mechanism for tRNA self-aminoacylation. In case a pair of codon and AC triplets occupied the first three base pairs of the proto-tRNA acceptor stem, their distinct electrostatic landscape would attract the cognate amino acid via the stereochemical affinity. Accommodation of an amino acid in this “nest” could then enable self-aminoacylation via folding back of the adjacent 3′ end tail, similar to the process shown to take place in a small ribozyme having a 3′ tail of four nucleotides [20]. The only current report of non-enzymatic aminoacylation of tRNAs is concerned with charging tRNA^Phe^ under high pressure, in the absence of ATP and synthetase [21]. It is therefore possible that the prebiotic self-aminoacylation process may have required high pressure as well, such as would prevail in submarine hydrothermal vents that were associated with the origin of life on earth [22]. This self-aminoacylation mechanism requires only a “soft“ version of Woese’s stereochemical affinity. It suffices that a certain amino acid would have higher affinity towards its cognate coding triplets, compared to that exhibited by the limited number of the other contemporaneous amino acids, to allow the formation of conceivable percentage of correctly aminoacylated tRNAs, thus- of correctly translated proteins.

With this hypothetical self-aminoacylation mechanism in mind, a large scale analysis of tRNA sequence data took place, in search for vestiges of cognate coding triplets in the acceptor-TΨC stems of the 20 proteinogenic tRNAs [9]. Surprisingly, the search revealed extreme occurrence of coding triplets, far beyond the statistical expectations, in the 3′ side of the acceptor-TΨC stems from the bacterial tRNAs of nine amino acids (Figure S1). The cognate coding triplets observed in the acceptor stems of Gly, Pro, Ser, Ala and His were specifically located in positions 68–70 and 70–72 (Figure 2 and Figure S1), positions which are referred to as “conservation sites”. The occupancy of the conserved coding triplets at the conservation sites of the first three amino acids is almost 100%, while the reduced occurrence observed in Ala and His data, where only about 65% of their sequences carry the conserved coding triplet in the conservation site (Figure S1), was attributed to atypical alterations in their modern charging process [9]. Ala, Gly, Ser and Pro are widely held to be among the first amino acids to emerge [23]. They could have therefore participated in such a primordial self-aminoacylation process, whereby, by being accommodated on the stem coding triplets of their cognate proto-tRNA, they would be non-enzymatically esterified to its 3′ end.

Later, when specific synthetases emerged, the stem coding triplets would cease to be directly involved in aminoacylation, but could have constituted the template for establishing the mutual recognition scheme between the primordial synthetase and its cognate proto-tRNA. This initial recognition mode is assumed to be still carried out by the modern synthetases of Ala, Gly, His, Pro and Ser, that belong to class IIa [10], accounting for the extreme evolutionary preservation of coding triplets in the corresponding acceptor stems. The initial synthetase would have thus likely to be the ancestor of the modern class II synthetases [2,6], or specifically of class IIa [9,24], which use the flexible motif 2 loop for tRNA identification (Figure 3b). Preferred accessibility to the corresponding motif 2 loop may have determined the location of conserved coding triplets in the acceptor stem. In the case of Ser, in particular, the exceptionally long motif 2 loop of SerRS allows it to interact with the whole 68–72 range [25,26], consistent with the conservation of codons in positions 68–70 as well as in 70–72 (Figure 2 and Figure S1).

The stem coding triplets suggested to be serving as modern aminoacylation identity determinants (Figure S1), i.e., GCU for Ala, CCC and UCC for Gly, CAC for His, CCG for Pro and UCA, UCC, UCU for Ser, are assumed here to be the fundamental triplets that initiated the genetic code of these amino acids. This subgroup would have been later supplemented, mostly via random mutations of the wobble nucleotide, resulting in their current degenerate codon set.

### 2.3. Autonomous Formation of Proto-tRNAs

The previous sections imply that the proto-tRNA involved in the initial stage of primordial translation should have already been an L-shaped molecule with a cloverleaf-like secondary structure. Additionally, it should carry a cognate pair of codon-AC triplets in the first three base pairs of the acceptor stem, an AC triplet in the anticodon loop and have a 3′ end tail that would enable self-aminoacylation. The feasibility of autonomous formation of such proto-tRNAs in the chemistry era, entities which could have turned functional with the advent of an initial living system, is examined.

#### 2.3.1. Formation of a 3-Arm Proto-tRNA

**Figure 4:**
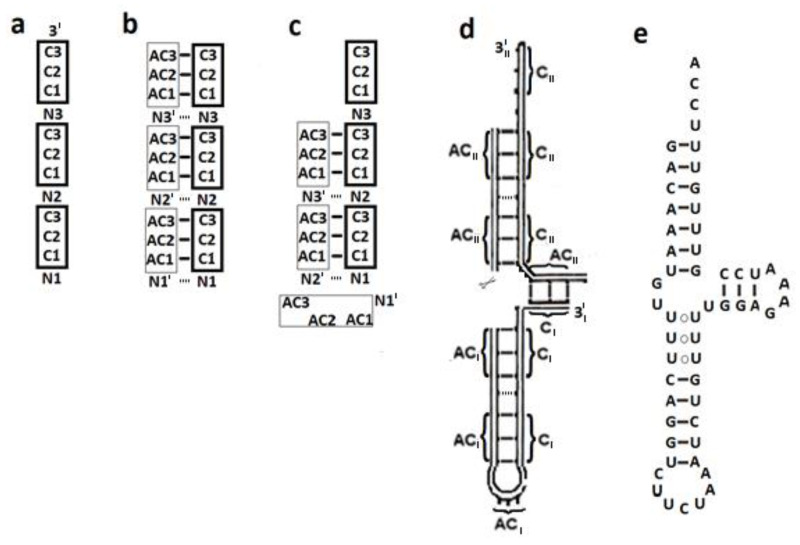
Model for the spontaneous formation of a 3-arm proto-tRNA (**a**) the initial 12-mer RNA strand carries three copies of the codon C_1_C_2_C_3_ (in thick frame). N signifies a nonspecific nucleotide. (**b**) Condensation of a complementary strand (AC triplets in thin frame) (**c**) Unzipping of the duplex and reassociation in a shifted manner yields a hairpin carrying corresponding coding triplets in the stem and in the loop. Throughout, solid lines represent Watson-Crick base pairs and dashed lines—potential base pair occurring when nonspecific nucleotides accidentally complement. (**d**) Base pairing between two hairpins associated with the same amino acid, marked by I, II, yields the 3-arm model. Disconnected points on the outer line symbolize points of ligation of the original 12-mer strands. (**e**) Compatibility with the mitochondrial tRNA^Ser^ from Ascaris suum [27].

The autonomous formation of a hairpin with a 3′ end tail of four nucleotides, which carries a codon triplet in its stem and a corresponding AC triplet in its loop, can be accomplished via a simple process. An 8-mer oligonucleotide that contains a specific codon in positions 2–4 and 6–8, with nonspecific nucleotides separating them, could serve as a template for the condensation of a complementary 8-mer strand. Detachment and reassociation of the two strands in a shifted manner would yield a three base pair hairpin with the aforementioned characteristics. This process is described in Figure 4a–c, but starting from a 12-mer RNA string that corresponds to the size of the modern cloverleaf tRNA structure. This string carries three copies of a particular nucleotide triplet, separated by nonspecific nucleotides (Figure 4a).

The probability of encountering such a 12-mer string is (1/4)^6^, that is, more than 0.02% of the RNA strands in a pool of random 12-mer oligonucleotides, will display such a pattern. The original 12-mer strand could serve as a template for the non-enzymatic condensation of a complementary strand (Figure 4b) and separation of the hybridized duplex via alterations of environmental factors ([28] and references therein), could enable the codon-only strand and its replicate, the AC-only strand, to re-associate in a shifted manner (Figure 4c). A single ligation between N2′ and N2 would then result, depending on the random complementarity between the nonspecific nucleotides, in a hairpin of six or seven base-pairs. This hairpin will contain an AC triplet in its loop and a codon-AC pair in the stem, adjacent to a 3′ end tail of four nucleotides, having therefore the potency to be self-aminoacylated. However, such an aminoacylated hairpin would be unfit to participate in translation, due to the absence of a second arm and it is not clear whether this hairpin, or shorter ones obtained via a similar process, could have had any prebiotic role.

In order to take part in translation, the proto-tRNA would be required to be L-shaped. This is achievable via the hybridization of two such hairpins, associated with the same amino acid. Base-pairing between the codon on the 3′ end of one hairpin and the AC triplet obtained from the anticodon loop of the second hairpin, subsequently to a cut made next to its 5′ (Figure 4d), would have yielded a simplified cloverleaf-like structure that misses the D-arm. This 3-arm proto-tRNA model is analogous in shape and in size to the modern mitochondrial tRNA that misses the D-stem (Figure 4e). It is composed of two codon-only and two AC-only 12-mer strands, holds an AC triplet in its anticodon loop suitable for decoding the proto-mRNA, and a codon-AC pair, which could have enabled self-charging. This proto-tRNA model therefore contains the structural elements required for participating in the initial prebiotic translation, while the T-loop, D-arm and the variable region (Figure 1a) would have evolved later.

#### 2.3.2. Formation of the 4-Arm Proto-tRNA

The autonomous formation of a complete cloverleaf secondary structure is achievable via the congregation of three 12-mer codon-only strands and three AC-only strands. In this case the two types of strands have a nonspecific nucleotide at their 5′ end, meaning that only 11 out of the 12 nucleotides are complementary.

**Figure 5:**
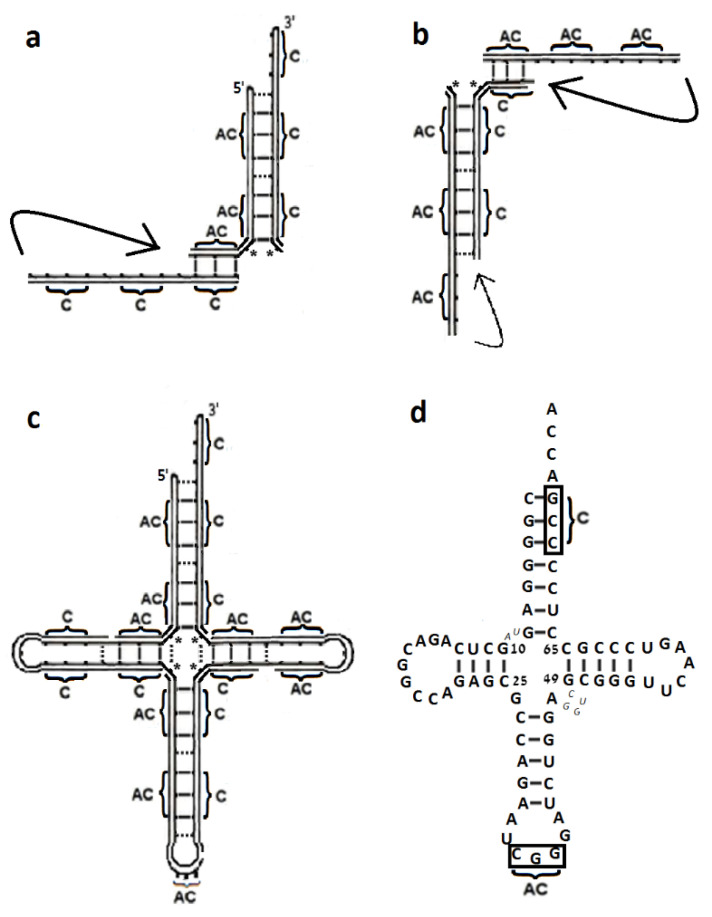
Model for the spontaneous assembly of a 4-arm proto-tRNA: (**a**) two 12-mer codon-only strands, base paired to a single AC-only strand, generate a 2-arm entity. Optional loops, formed via a single ligation, are marked by arrows. (**b**) As in (**a**) but composed of two AC-only and a single codon-only strand. Non-specific nucleotides acting as “snap fasteners” that can combine (**a**,**b**) into the 4-arm scheme are depicted by asterisks. (**c**) Formation of a 4-arm cloverleaf scheme, compatible with the modern tRNA. (**d**) Secondary scheme of tRNA^Pro^ from Thermotoga maritima [27]. The cognate coding triplet CCG, found in positions 70–72 in 98% of the acceptor stems from bacterial tRNA^Pro^ and in the anticodon loop, is marked. Pseudouridine in the T-stem is referred to as U. Nucleotides lacking counterparts in the 4-arm model are indicated by smaller italic letters.

A 2-arm RNA entity can assemble spontaneously through base pairing between a single AC-only strand and two codon-only strands (e.g., Figure 5a) and between a single codon-only strand and two AC-only strands (e.g., Figure 5b). Following the hybridization, each tail can fold into a loop via a single ligation. These 2-arm entities could have subsisted in the prebiotic environment, being, due to the base pairing, relatively stable and protected against cleavage. In case the non-specific nucleotide 10 in one 2-arm entity was complementary to the non-specific nucleotide 25 in a second 2-arm entity and nucleotide 49 was complementary to 65 (Figure 1a), they could have acted as “snap fasteners”, generating a 4-arm cloverleaf structure via base pairing (Figure 5a–c). The resulting secondary structure would contain 72 nucleotides, revealing impressive resemblance to the cloverleaf structure of the canonical tRNA (Figure 5d). The omission of nucleotides 8, 9 and 45–48 from the variable region would not affect the functional acceptor and anticodon arms, but the tRNA elbow region (Figure 1b) is likely to have been diverse to some extent and plausibly, simpler. The length of the 4 arms, the number of base pairs, the number of nucleotides in the three loops and the length of the 3′ end tail show close similarity to the modern tRNA (Figure 5c,d). A potential tertiary contact between the D- and T- loops, involved in stabilizing the L-shape of the modern tRNA, is guaranteed as well. The AC triplets that comprise the T-loop in the 4-arm model are complementary to the codon triplets comprising the D-loop (Figure 5c), thus allowing the formation of long-distance T-D base pairs (Figure 1a).

Assembling a proto-tRNA from three codon-only strands and their fully complementary replicates, i.e., three AC-only strands that have a nonspecific nucleotide at their 3′ instead of at the 5′, would have also led to a 4-arm model of 72 nucleotides that retains considerable equivalence to the modern cloverleaf secondary structure. However, the AC triplet in the anticodon loop will be off center (as occurs in Figure 4c) and one of the two “snap fasteners”, i.e., the base pair between nucleotides 10 and 25, which is engaged in assembling the final 4-arm model (Figure 1a and Figure 5c,d), will be lost, thus reducing the analogy to the modern tRNA. 

These 3-arm and 4-arm RNA entities could have been floating in the prebiotic environment, being positively selected due to the negative change in their free energy during folding, which lends tRNA-like molecules with significant stability, together with the enhanced protection against cleavage stemming from the base pairing scheme. Conditional on possessing the required dynamic properties, these initially-inert entities could have been later incorporated into the emerging translation system. Regardless of whether the earliest proto-tRNA was similar to the 3-arm model, which in contemporary biology is found only in about 1/3 of the mitochondrial tRNA^ser^, or to the 4-arm model, which is dominant nowadays, certain nucleotides forming elaborated tertiary interactions are assumed to join later. The identity of the nucleotides that constituted the initial array of coding triplets, and are currently not involved in recognition, could have been lost over time. On the other hand, the identity of the functional nucleotide-triplets, i.e., those of the AC triplet in the anticodon loop and those of the coding triplets in the bacterial acceptor stems of Ala, Gly, His, Pro and Ser, was highly retained through evolution.

#### 2.3.3. The 4-Arm Model vs. Coding-Triplets Conservation in the Modern Acceptor Stems

According to the present model, the first three base pairs of the proto-tRNA engaged in self-aminoacylation are expected to carry a pair of cognate codon-AC triplets. This requirement is met by the majority of the modern bacterial tRNAs of His, Pro and Ser (Figure 2 and Figure S1). However, the proposed model should account also for additional statistical occurrences observed in the data, i.e., conservation of coding triplets in positions 68–70, which are too distant to be involved in self-aminoacylation, and the correlated absence of coding triplets in positions 69–71 (Figure 2).

Both these observations can conform to the current model under two assumptions: 1. the conserved coding triplets that currently reside in positions 68–70, existed initially also in positions 70–72, where they served for self-aminoacylation. 2. These initial coding triplets were symmetrical, i.e., of the type—N_1_N_2_N_1_, or N_1_N_1_N_1_, where N is A, C, G or U. In this case, according to the model, the same nucleotide would occur at positions 68, 70 and 72 and if the nonspecific nucleotide at position 69 was accidentally identical to the nucleotide in position 71, an event whose statistical probability is 25%, a duplicate of the coding triplets in positions 70–72, would be found in positions 68–70, but not in positions 69–71. Indeed, in accordance with the model, most of the coding triplets conserved in positions 68–70 in tRNA^Ser^ (UCU) and tRNA^Gly^ (CCC) are symmetric (Figure S1). The initial coding triplets of Ala and Gly, which are assumed to have resided in positions 70–72 in the prebiotic self-aminoacylation stage, are not found in the modern tRNAs, likely due to subsequent sequence alterations that followed the emergence of the specific synthetases.

## 3. Discussion

The translation system stands at the hub of “life as we know it”. It is therefore necessary, in any scenario concerned with its advent through natural procedures, to present a mode by which a proto-ribosome and aminoacylated proto-tRNAs could have spontaneously materialized via standard chemical processes. Once formed, these RNA elements could have cooperated in translating codes embedded in random RNA chains into polypeptides, which, after folding, would fortuitously have had some catalytic abilities.

A vast bulk of studies from many disciplines, aimed at outlining a possible path from the inanimate material into life as we know it, have already achieved significant progress. The feasibility of spontaneous formation, under conditions assumed to prevail in the prebiotic world of nucleotides [29,30], of amino acids [31], of RNA chains longer than 100-mer [32,33], together with the capability of short oligonucleotides to serve as templates for the condensation of their complementary strands [34], and the ability of RNA duplexes to unravel spontaneously ([28] and references therein) were already verified in the lab. Additionally, a model for the autonomous formation of a non-coding proto-ribosome, derived from the symmetrical region enclosing the PTC of the contemporary large ribosomal subunit [35], which materialized via the dimerization of two L-shaped RNA entities, was suggested [36,37]. Recently such dimeric proto-ribosomes were experimentally demonstrated to assemble spontaneously, catalyzing peptide bond formation and yielding short peptides [38,39].

### 3.1. Self-Aminoacylation and the Fundamental Code

Carl Woese’s stereochemical affinity hypothesis [19], taken together with the extreme occurrence of cognate coding triplets in the acceptor stems from the bacterial tRNAs of several ancient amino acids [9] (Figure 2), yield a novel approach to two major issues concerned with the origin of translation, i.e., the origin of the genetic code and the prebiotic aminoacylation of proto-tRNAs. Enhanced affinity of certain amino acids towards a specific nucleotide triplet located in positions 1–3:70–72 of an autonomously formed proto-tRNA, is suggested here to underlie the emergence of the standard genetic code, while the proposed mechanism for self-aminoacylation of proto-tRNAs resolves the chicken and egg conundrum concerned with the spontaneous advent of enzymatic aminoacylation in the prebiotic era.

The verity of the stereochemical affinity, which forms the basis for the self-aminoacylation process, requires confirmation. Up till now it was tested mainly on aptamers [40] and within the ribosome [41], giving inconclusive results [23]. However, initial docking simulations seem to indicate an enhanced preference of few amino acids towards their cognate coding triplets located in the acceptor stem (unpublished results). The present hypothesis utilizes a “soft” version of the stereochemical affinity, i.e., one which requires that amino acids whose proto-tRNAs took part in self-aminoacylation would have had higher affinity towards their cognate coding triplets, relative to that exhibited by the coexisting amino acids towards the same trinucleotide. Conditional on that, when a coding triplet accessible to the back-folded 3′ end tail inhabited an amino acid, self-aminoacylation could have taken place, yielding mainly correctly charged aa-tRNAs, thus offering a realistic chance for obtaining correctly translated RNA strands. 

Separate elements from the linkage made here between the origin of coding and a prebiotic self-aminoacylation process, were already put forward many years ago. The existence of a specifically fitted pocket in the acceptor stem, suitable for accommodating amino acids, was suggested by Woese [42]. Noller [8], more particularly, assumed that the acceptor-end of the proto-tRNA would have had recognition capability, via direct amino acid-RNA interaction, possibly involving a codon-AC duplex. This interaction, however, was not linked with self-aminoacylation. Rodin and Ohno [43], similarly proposed recognition via codon-AC-like pair located at the first positions of the acceptor stem, but it referred to the enzymatic charging of proto-tRNAs by an ancient synthetase and not to an earlier non-enzymatic aminoacylation. De Duve [44] suggested the existence of “proto-paracodons”, i.e., of stem identity determinants that depended on a stereochemical interaction with their cognate amino acids, preceding the emergence of the proto-synthetases. De Duve’s paracodons, however, referred to discrete identity determinants that constituted a second genetic code, while the recent analysis demonstrated that all but one of these stem identity determinants, i.e., all but one of the paracodons of Ala, Gly, His, Pro, and Ser are contained within their modern cognate coding triplets. In other words, it indicated that the second genetic code located at the acceptor stem actually makes part of the standard genetic code [9]. Crick [45] went even further and referred to self-aminoacylation of the initial proto-tRNAs as “an attractive idea (suggested by Dr. Oliver Smithies) … that the primitive tRNA was its own activating enzyme. That is, that its structure had a cavity in it which specifically held the sidechain of the appropriate amino acid in such a position that the carboxyl group could be easily joined on to the terminal ribose of the tRNA”. Crick, nevertheless, did not particularly link the acceptor stem cavity that enabled self-aminoacylation with the stereochemical affinity of amino acids towards their cognate stem coding triplets, as done here.

It is possible that at the initial stage of life advent, merely the proto-tRNAs of Ala, Gly, His, Pro, and Ser took part in translation, because only these five amino acids portray an ordered pattern of coding-triplets in the acceptor stems of their modern tRNAs (Figure 2 and Figure S1). However, analysis of the tRNA sequence data (Figure S1), found excess of cognate coding triplets in the acceptor-TΨC stems of Asp, Arg, Leu, and Val as well, distributed in a manner that cannot be linked with contemporary aminoacylation [9]. This observation evokes the possibility that the proto-tRNAs of these four ancient amino acids [23] participated in the prebiotic self-aminoacylation as well, but their subsequent charging by synthetases that do not belong to class IIa, rendered their stem coding triplets unnecessary. In agreement, the coding triplets in the acceptor-TΨC arms of Arg, Leu, and Val display loss of specific positioning and identity, together with an overall reduction in their level of occurrence (Figure S1). A different evolutionary path is suggested by the data from the bacterial tRNA^Asp^, where the cognate coding triplet, GUC, is found in 91% of its sequences in positions 64–66, a conservation that was attributed to its involvement in the recognition by EF-Tu [9]. The synthetases of the remaining eleven amino acids, which are mostly late appearing amino acids [23], would add over time and their proto-tRNAs would skip the rudimentary self-aminoacylation stage. Consequently, their modern tRNAs lack conserved coding triplets in their acceptor stems altogether [9]. 

### 3.2. Models for the Spontaneous Formation of Cloverleaf tRNA

The indispensability of tRNAs in any scenario proposed for the emergence of the translation system elicited a plethora of schemes concerned with their autonomous formation [6,44,46,47,48,49,50,51,52,53,54]. Two central tenets regarding the assembly of a cloverleaf tRNA structure were suggested. The first, which is derived from the tertiary L-shape of the modern tRNA (Figure 1b), divides the molecule into an older part, the coaxial acceptor-TΨC half, and to a late appearing anticodon-DHU half [48,55]. The second approach assumes that the cloverleaf secondary structure of the proto-tRNA was obtained by the conjugation of two hairpins, analogous to the 3′ and 5′ halves of the modern tRNA. The two halves could have had complementary sequences, that is, the 3′ half acted as a template for generating the 5′ half [46]. Alternatively, the strands of the two hairpins would be obtained via duplication, i.e., had nearly identical sequences [49,50,51,52]. The assumed limited size of the primordial synthetase that permitted it to reach only recognition elements residing in the acceptor stem [4,7,8], pointed to the functional importance of having coding information in the vicinity of the 3′ end, and this requirement was incorporated in many schemes suggesting the formation of the proto-tRNA [4,6,15,43,44,45,50,53]. 

The first approach carries inherent difficulties. It assumes that the initial proto-tRNA engaged in translation was an aminoacylated coaxial acceptor-TΨC helix, a possibility that can be ruled out due to the spatial requirement for a second arm (Figure 3a) [16]. Moreover, the corresponding coding triplets found in the acceptor stem and in the anticodon loop of modern tRNAs belonging to early emerging amino acids (e.g., Figure 5d) cannot be explained by this proposition. When referring specifically to the first tRNA-like molecules assumed to emerge autonomously in the inanimate world, the second approach is unlikely as well. It requires the accidental occurrence of two sequence-related RNA strands of about 40-mer, being either complementary [46] or nearly identical [49,50,51,52]. Accidental occurrence of two corresponding sequences of such length, that would conjugate to form the cloverleaf secondary structure, is extremely unlikely. An alternative path, which proceeds through the replication or duplication of an original 40-mer strand in the absence of a replicase, followed by unwinding of this long helix to form the two hairpins that would later combine into a cloverleaf proto-tRNA, seems dubious as well. Indeed, the statistical probability of such formation schemes is generally bypassed, except in Nagaswamy and Fox [51], where a probability of 1 in 30 million for the occurrence of a random sequence suitable for the formation of a cloverleaf tRNA structure is computed, a figure which is low, but still realistic. However, with the advent of a replicating molecule, the cumbersome self-assembly of proto-tRNAs, suggested here to occur in the chemistry era, would be substituted by a simpler process, likely involving replication, as suggested by the second approach. Such a mechanism is supported by the genome of Nanoarchaeum equitans, the smallest and simplest thermophilic archaea known today, which creates functional tRNAs from separate genes for their 5′- and 3′- halves [56].

The schemes presented here for spontaneous formation of the 3-arm and 4-arm L-shaped tRNAs, which carry coding information both in their anticodon loop and in positions 1–3:70–72 of the acceptor stem, are novel. The models are assembled from 12-mer RNA strands carrying an array of three coding triplets, a pattern that is still recognizable in the sequences of bacterial tRNA^Arg^, where poles of occurrence appear in positions 66–68 and 70–72 (Figure S1), while the third coding triplet expected according to the model, in positions 74–76, is exchanged by the universally conserved CCA. In spite of the simplicity and statistical feasibility of the schemes, these models are not flawless. The 3-arm model results in an off-center positioning of the AC triplet in the anticodon loop (Figure 4c), which might have obstructed base pairing with the earliest mRNA. The shape of the 4-arm model is highly compatible with that of the modern tRNA (Figure 5c,d), but accumulation of its building blocks in a prebiotic site, i.e., of the 12-mer codon-only and AC-only strands, would have been less-trivial, because only 11 out of the 12 nucleotides are fully complementary.

In spite of the aforementioned weaknesses, the present models for the proto-tRNA non-catalyzed formation seem to provide substantial advantages over previous hypotheses:

(a) The models require an initial RNA strand with an array of three coding triplets that can be found in more than 0.02% of the 12-mer oligonucleotides with random sequences. Adding to that the requirement that the non-specific nucleotides forming the “snap fasteners” should be complementary (Figure 5c,d), results in a statistical probability of (1/4)^8^, i.e., about 1 in 70,000 random 12-mer oligonucleotides would be suitable for serving as a building block of a cloverleaf proto-tRNA.
(b) Spontaneous replication of 12-mer RNA strands, their unzipping under change in environmental conditions and recombination to form the 3-arm or 4-arm models, are feasible chemical reactions in a prebiotic environment lacking biological catalysts.
(c) Self-assembly of the strands relies on base pairing, a reaction which occurs spontaneously. The few ligation reactions required for forming the loops in the cloverleaf model are facilitated by the proximity of the ligated nucleotides, whose strands are already held together by base pairing.
(d) The 4-arm model preserves the primary and secondary constraints that underlie the three-dimensional folding of the tRNA structure; it naturally yields a 3′ end tail of four nucleotides, allows tertiary base pairing between the D–T loops, and guarantees the existence of coding triplets at the beginning of the acceptor stem and in the anticodon loop.
(e) The 3-arm and 4-arm tRNA models closely resemble, both in size and in their secondary structures, the tRNAs participating in contemporary translation (Figure 4d,e and Figure 5c,d).
(f) The formation of cloverleaf tRNA structure from 12-mer oligonucleotides can be experimentally examined. Placing in a test tube, under various environmental conditions, 12-mer RNA strands, each carrying an array of three codons or of the complementary three AC triplets, with appropriate “snap fasteners” nucleotides, is suggested here to enable the spontaneous formation of some tRNA molecules with a cloverleaf secondary structure.

### 3.3. Was There an RNA World?

The difficulty to envisage a process that could have led, entirely via natural means, from an inanimate world dominated by chemical reactions, into the complexity of “life as we know it”, invoked a plethora of hypotheses suggesting that cooperativity between primordial, self-emerging molecules, could have generated simple living systems. The most popular and appealing hypothesis, the “RNA world” [42,45,57], was based on the idea that abiotically synthesized RNA strands could have acted as both the genetic material and the catalysts, to generate a primordial form of life which possessed properties of multiplication, variation, and heredity. This set of RNA molecules, however, could not have continuously evolved into LUCA, because its replicase would be made of RNA, while in “life as we know it” only enzymes, the polymerases, replicate nucleic acid chains. It follows that even if an RNA world did exist, preceding the earliest version of “life as we know it”, a replicating enzyme, i.e., a proto-polymerase, would have to emerge from scratch at some point, totally disengaged from the activity in the RNA world encapsulating it, to replace the replicase. Such an evolutionary discontinuity seems unlikely.

An alternative view would posit the initial steps out of the chemistry era in an RNA-protein world, where RNA and proteins cross-catalyzed the formation of each other, conforming to the principle of molecular mutualism [58]. The initial, minimalist, self-emerging set of this type, which could have continuously evolved into the key part of “life as we know it”, i.e., into the translation system, should have included a relatively simple version of a processive ribosome, aminoacylated proto-tRNA molecules, a proto-polymerase as well as free amino acids and nucleotides. Previous studies suggested a feasible path to the spontaneous emergence of a simple proto-ribosome [16,35,36,37,38,39]. Here mechanisms that could have enabled, already in the chemistry era, the autonomous formation of cloverleaf proto-tRNAs and their specific aminoacylation, are put forward. The proto-ribosome, possibly stabilized by random peptides, together with these aminoacylated proto-tRNAs, could have cooperated, translating arbitrary RNA chains into random polypeptides via a factor-free protein synthesis mechanism, generating, in rare cases, proteins possessing weak catalytic abilities of some sort. If once, in a single, extremely rare event, the translation of a random RNA chain yielded a protein with weak polymerase activity, a molecular set that could have continuously evolved into the translation system of LUCA would be completed [59]. The feasible advent of aminoacylated proto-tRNAs and of proto-ribosomes, that could have prompted the emergence of an RNA-protein world, makes the “RNA world” redundant, and lays the foundation for a prebiotic system operating according to the central dogma of biology.

Carl Woese wrote “look to the tRNAs for the answer. tRNA is … a central component of the translation mechanism to begin with and it defines the mechanism still to this day“ (Woese C., private communication). The hypotheses presented here, concerned with the self-assembly of the tRNA, its self-aminoacylation, and the emergence of the genetic code, seem to provide a feasible starting point, that when combined with the accumulating information about various aspects of the origin of life, may promote a feasible scenario for life emergence via natural processes.

## Data Availability

The current study refers to data published in reference [9].

## Supplementary Materials

The supporting information can be downloaded at: https://www.mdpi.com/article/10.3390/ijms232415756/s1.
