# Supplementary material for: Prebiotic Assembly of Cloverleaf tRNA, Its Aminoacylation and the Origin of Coding, Inferred from Acceptor Stem Coding-Triplets
Source: Int J Mol Sci. 2022 Dec 12;23(24):15756. doi: 10.3390/ijms232415756 (PMC9778954; doi:10.3390/ijms232415756)
Supplement: Supplementary file 1 [file ijms-23-15756-s001.zip › ijms-2021046-supplementary.pdf]

Figure S1

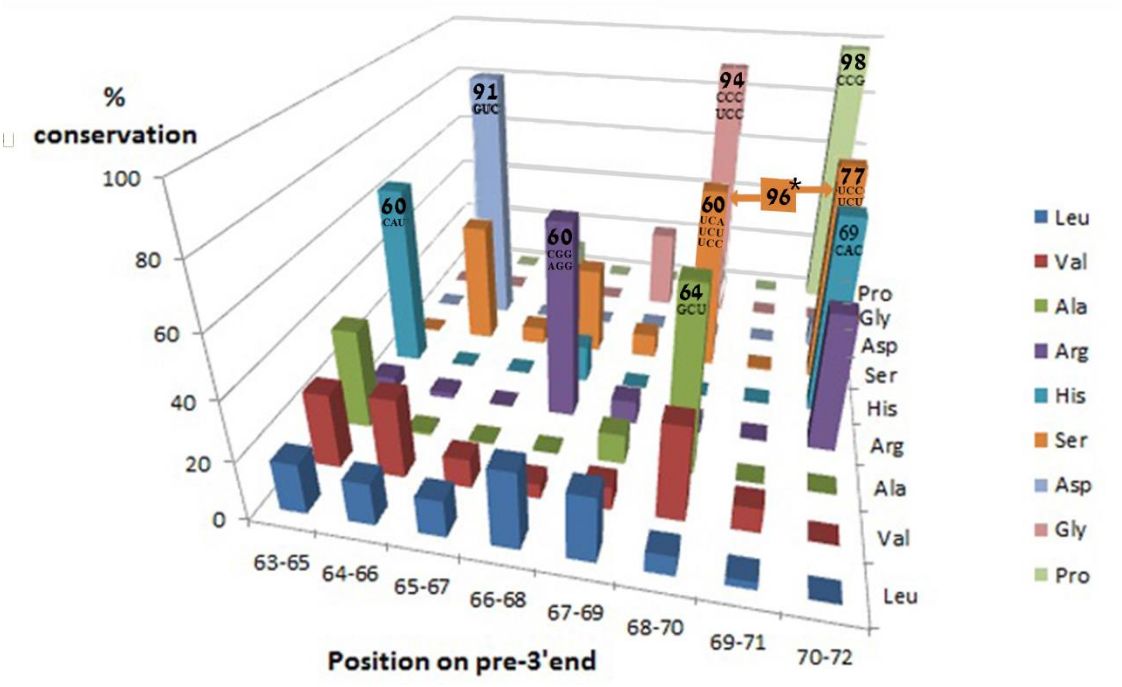

Figure S1: **Conserved coding triplets in positions 63-72** in the tRNAs of the nine conserving amino acid (Agmon et al. 2021). Each column is marked by the identity of its conserved triplets and by the percentage of sequences carrying these triplets in the site.

* The overall occurrence of the conserved coding triplets of Ser in its two partially overlapping conservation sites (positions 68–70 and 70–72).

Reference:

Agmon I, Fayerverker I, Mor T. Coding triplets in the tRNA acceptor-TΨC arm and their role in present and past tRNA recognition. *FEBS Lett*. 2021, **595**, 913-924.
